# Unintended targeting of *Dmp1-Cre* reveals a critical role for Bmpr1a signaling in the gastrointestinal mesenchyme of adult mice

**DOI:** 10.1038/boneres.2016.49

**Published:** 2017-01-31

**Authors:** Joohyun Lim, Joseph Burclaff, Guangxu He, Jason C Mills, Fanxin Long

**Affiliations:** 1 Department of Orthopaedic Surgery, Washington University School of Medicine, St. Louis, MO, USA; 2 Division of Gastroenterology, Departments of Medicine and Pathology and Immunology, Washington University School of Medicine, St. Louis, MO, USA; 3 Department of Orthopedics, The Second Xiangya Hospital, Central South University, Hunan 410011, China; 4 Department of Developmental Biology, Washington University School of Medicine, St. Louis, MO, USA

## Abstract

Cre/loxP technology has been widely used to study cell type-specific functions of genes. Proper interpretation of such data critically depends on a clear understanding of the tissue specificity of Cre expression. The *Dmp1-Cre* mouse, expressing Cre from a 14-kb DNA fragment of the mouse *Dmp1* gene, has become a common tool for studying gene function in osteocytes, but the presumed cell specificity is yet to be fully established. By using the *Ai9* reporter line that expresses a red fluorescent protein upon Cre recombination, we find that in 2-month-old mice, *Dmp1-Cre* targets not only osteocytes within the bone matrix but also osteoblasts on the bone surface and preosteoblasts at the metaphyseal chondro-osseous junction. In the bone marrow, Cre activity is evident in certain stromal cells adjacent to the blood vessels, but not in adipocytes. Outside the skeleton, *Dmp1-Cre* marks not only the skeletal muscle fibers, certain cells in the cerebellum and the hindbrain but also gastric and intestinal mesenchymal cells that express *Pdgfra*. Confirming the utility of *Dmp1-Cre* in the gastrointestinal mesenchyme, deletion of *Bmpr1a* with *Dmp1-Cre* causes numerous large polyps along the gastrointestinal tract, consistent with prior work involving inhibition of BMP signaling. Thus, caution needs to be exercised when using *Dmp1-Cre* because it targets not only the osteoblast lineage at an earlier stage than previously appreciated, but also a number of non-skeletal cell types.

## Introduction

Dentin matrix protein 1 (DMP1) is an extracellular phosphorylated glycoprotein belonging to the SIBLING (small integrin-binding ligand N-linked glycoprotein) family of proteins.^1^ Originally discovered in the dentin matrix, DMP1 is also highly expressed in other mineralized tissues including bone and cartilage.^2–4^ Functional studies have demonstrated important functions of DMP1 in regulating not only biomineralization but also phosphate homeostasis in both mice and humans.^5–8^ Expression of DMP1 has also been detected in a variety of non-mineralizing tissues in the mouse, these including brain, liver, muscle, kidney, and pancreas, but its function there is not known.^9^

Cre/loxP technology enables gene deletion in specific cell types and thus allows for interrogation of gene function in a cell type-specific manner. Conditional deletion in specific lineages depends on the unique expression pattern of the Cre recombinase. The *Dmp1-Cre* transgenic mouse line was generated to express Cre from a 14-kb promoter fragment (−9 624 to +4 439) of the mouse Dmp1 gene.^10^ The promoter encompassed a 9 624-bp promoter, the 95-bp exon 1, the 4 326-bp intron I plus the 17-bp initial noncoding sequence of exon II. The initial characterization of the mouse line with the Rosa26R mouse (expressing β-galactosidase upon Cre recombination) identified strong Cre activity in osteocytes and odontoblasts but not osteoblasts.^10^ However, detection of β-galactosidase expression relied on an enzymatic reaction *in vitro* known to be susceptible to tissue preparation and reaction conditions. In fact, a more recent analysis of *Dmp1-Cre* with a reporter mouse expressing a fluorescent protein revealed Cre activity in additional cell types besides osteocytes, most notably skeletal muscle and osteoblasts.^11^ The study also implicated cells within the bone marrow and those in the brain, but did not provide a detailed description. Thus, a systematic survey of tissues targeted by *Dmp1-Cre* in the mouse is warranted.

Much work has been done to decipher the contribution of BMP signaling to gastrointestinal development and maintenance, but the specific role of BMP reception by mesenchymal tissue remains unclear. In the stomach, Mx1-Cre-mediated *Bmpr1a* deletion resulted in polyp formation at the esophageal and antral transition zones.^12^ Similarly, *Bmpr1a* removal by the ubiquitous inducible CAGGCreER driver cause dantral polyps and antral-pyloric hyperplasia.^13^ Conversely, overexpression of the secreted BMP antagonist Noggin in parietal cells and intestinal villi caused gastric cysts and intestinal polyps, respectively.^14,15^ However, when BMP signaling was selectively disrupted in the intestinal epithelium through deletion of *Bmpr1a*, no polyps formed despite increased proliferation and altered morphology within the epithelium.^16^ On the other hand, stromal deletion of Bmpr2 with nestin-Cre led to colorectal epithelial overgrowth and polyp formation, but the interpretation there was complicated by the fact that nestin-Cre targets multiple lineages including the epithelium.^17^ Overall, BMP signaling within the mesenchymal compartment likely contributes to normal gastrointestinal development and maintenance, but this notion warrants further investigation.

Here we assess the cell types targeted by *Dmp1-Cre* in 2-month-old mice by monitoring the expression of a red fluorescent protein (dtTomato) from the *Ai9* reporter allele. Consistent with previous findings, *Dmp1-Cre* targets not only osteocytes but also osteoblasts and preosteoblasts, along with a subset of bone marrow stromal cells, as well as the skeletal muscle and certain brain cells. Unexpectedly, *Dmp1-Cre* selectively targets gastrointestinal mesenchymal cells with high efficiency. Deletion of *Bmpr1a* with *Dmp1-Cre* results in polyposis throughout the stomach and intestines, demonstrating a critical role of mesenchymal BMP signaling in maintaining a normal gastrointestinal tract.

## Materials and Methods

### Mouse strains

*Dmp1-Cre*, *Ai9*, and *Bmpr1a^f/f^* mouselines are as previously described.^10,18,19^ *Ai9* mice were purchased from the Jackson Laboratory (Bar Harbor, ME, USA); *Dmp1-Cre* and *Bmpr1a^f/f^* mice were generously provided by Dr Jian Q Feng (Baylor College of Dentistry) and Dr Yuji Mishina (University of Michigan), respectively. Littermate mice with the genotype of *Dmp1-Cre*; *Bmpr1a^f/f^* (CKO) or *Bmpr1a^f/f^* (control) were generated by breeding the two genotypes as previously produced.^20^ The mice were in a mixed genetic background between C57BL6 and 129 strains. Both males and females were analyzed with similar results. All mouse procedures used in this study were approved by the Animal Studies Committee at Washington University.

### Cryostat sections

Two-month-old mice were perfused with 4% paraformaldehyde (PFA) as described previously.^21^ After perfusion, tibias were dissected and fixed in 4% PFA at 4 °C overnight. The fixed tibias were decalcified in 14% EDTA (pH 7.4) for 3 days, incubated in 30% sucrose at 4 °C overnight and then snap-frozen in optimal cutting temperature (OCT) embedding medium. Frozen sections were cut at 8 μm thickness with a cryostat equipped with CryoJane (Leica, Buffalo Grove, IL, USA). The sections were kept at −20 °C until analyses.

### Immunofluorescence staining

For detection of Pdgfra, perilipin, or endomucin, immunostaining was performed on cryostat sections using mouse polyclonal Pdgfra antibody (1:100; R&D Systems), or rabbit monoclonal perilipin antibody (1:100; Cell Signaling Technology, Danvers, MA, USA), or rat monoclonal endomucin antibody (1:100, Santa Cruz, Biotechnology, Dallas, TZ, USA). The secondary antibodies are as follows: Alexa Fluor 488 goat anti-mouse IgG (for Pdgfa); Alexa Fluor 488 goat anti-rabbit IgG (for perilipin), and Alexa Fluor 488 goat anti-rat IgG (for endomucin) (all at 1:500, Life Technologies, Grand Island, NY, USA). Sections were mounted with VECTASHIELD Mounting Medium containing DAPI (Vector Laboratories, Burlingame, CA, USA). Images were acquired with a Nikon confocal microscope (Melville, NY, USA).

### Analyses of the gastrointestinal tract

For proliferation assays, mice were injected intraperitoneally with 5-bromo-2’-deoxyuridine (BrdU, 120 mg·kg^−1^) and 5-fluoro-2’-deoxyuridine (12 mg·kg^−1^) in sterile water 90 min before killing. Following killing, stomachs were immediately excised and flushed with phosphate-buffered saline then inflated with freshly prepared formalin (10% formaldehyde, Sigma, St. Louis, MO, USA) in phosphate-buffered saline and the pylorus clamped with a hemostat. Inflated stomachs and segments of the small and large intestines were allowed to fix overnight in 10% formalin then transferred to 70% ethanol. Tissues were arranged in 3% agar in a tissue cassette, underwent routine paraffin processing, and 5 μm sections were cut and mounted on glass slides. For immunohistochemistry, sections underwent a standard deparaffinization and rehydration protocol then were blocked with 5% horse serum for 1 h before staining for BrdU using Goat anti-BrdU (1:20 000, gift of Dr Jeff Gordon, Washington University) and biotinylated horse anti-goat (1:200, Vector Laboratories) antibodies. Images were acquired using a Nanozoomer Slide Scanner (Hamamatsu, Japan, model 2.0-HT).

## Results

### *Dmp1-Cre* targets osteoblast-lineage cells, skeletal muscle, and bone marrow perivascular cells

**Figure 1:**
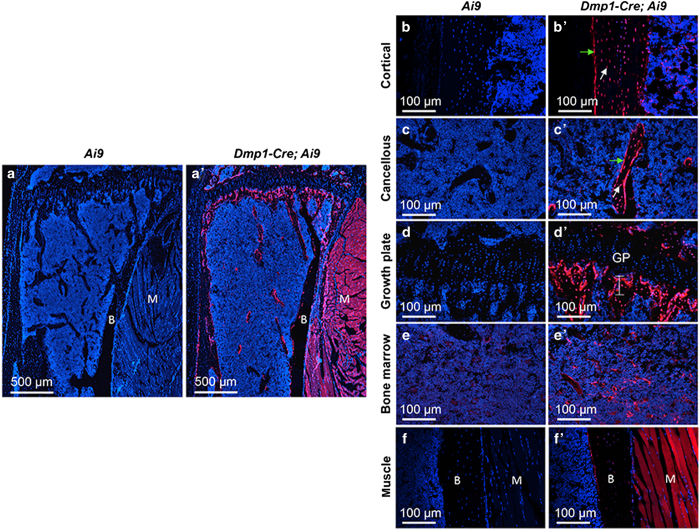
*Dmp1-Cre* targets osteoblast lineage cells, skeletal muscle, and bone marrow cells in 2-month-old mice. (**a**,**a**’) Confocal microscopy images of direct fluorescence from tdTomato on longitudinal sections of the proximal tibia. (**b**–**f**’) Images at a higher magnification for different areas of bone as indicated. B, bone; M, skeletal muscle; GP, growth plate. Green arrow, osteoblast; white arrow, osteocyte. Line in (**d**’) denotes chondro-osseous junction.

**Figure 2:**
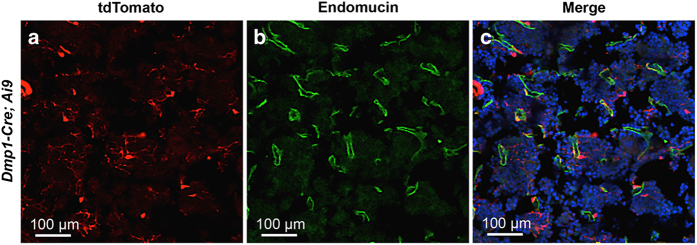
*Dmp1-Cre* targets bone marrow cells near blood vessels. Immunostaining against endomucin and direct fluorescence of tdTomato on sections of the tibial bone marrow from 2-month-old *Dmp1-Cre*; *Ai9* mice. (**a**) tdTomato; (**b**) endomucin; (**c**) merged image.

**Figure 3:**
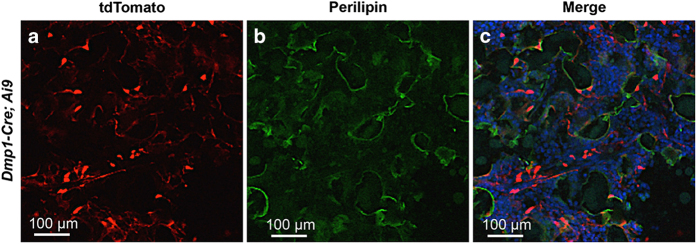
*Dmp1-Cre* does not target bone marrow adipocytes. Immunostaining against perilipin and direct fluorescence of tdTomato on sections of the tibial bone marrow from 2-month-old *Dmp1-Cre*; *Ai9* mice. (**a**) tdTomato; (**b**) perilipin; (**c**) merged image.

To characterize the targeting specificity of *Dmp1-Cre*, we generated *Dmp1-Cre*; *Ai9* mice (one copy each of *Dmp1-Cre* and *Ai9*) and analyzed tdTomato expression on sections of the limbs at 2 months of age. As expected, limb sections from the control *Ai9* mice did not exhibit any red fluorescence (Figure 1a-f), but those from *Dmp1-Cre*; *Ai9* mice showed strong signals both in the long bone and in the adjacent skeletal muscle (Figure 1a’–f’). Targeting of the skeletal muscle was not previously reported, but was detected here in all muscle fibers (Figure 1f’). Within the long bone, *Dmp1-Cre* marked not only osteocytes but also osteoblasts in both cortical and cancellous bone (Figure 1b’ and c’). In addition, the chondro-osseous junction immediately below the growth plate, an area enriched in preosteoblasts, expressed a strong signal even though the growth plate was negative (Figure 1d’). Red fluorescence was also detected in certain cells within the bone marrow, although generally at a lower intensity than those other cell types described above (Figure 1e’). Co-immunostaining experiments revealed that the red fluorescence-positive marrow cells were perivascular as they showed close proximity to the endothelium-expressing endomucin (Figure 2). On the other hand, perilipin staining showed that the bone marrow adipocytes were not targeted by *Dmp1-Cre* and generally did not show a close association with the targeted cells (Figure 3). Thus, in addition to osteocytes, *Dmp1-Cre* targets early-stage osteoblast-lineage cells, bone marrow perivascular cells as well as the skeletal muscle.

### *Dmp1-Cre* targets brain cells as well as gastrointestinal mesenchymal cells

**Figure 4:**
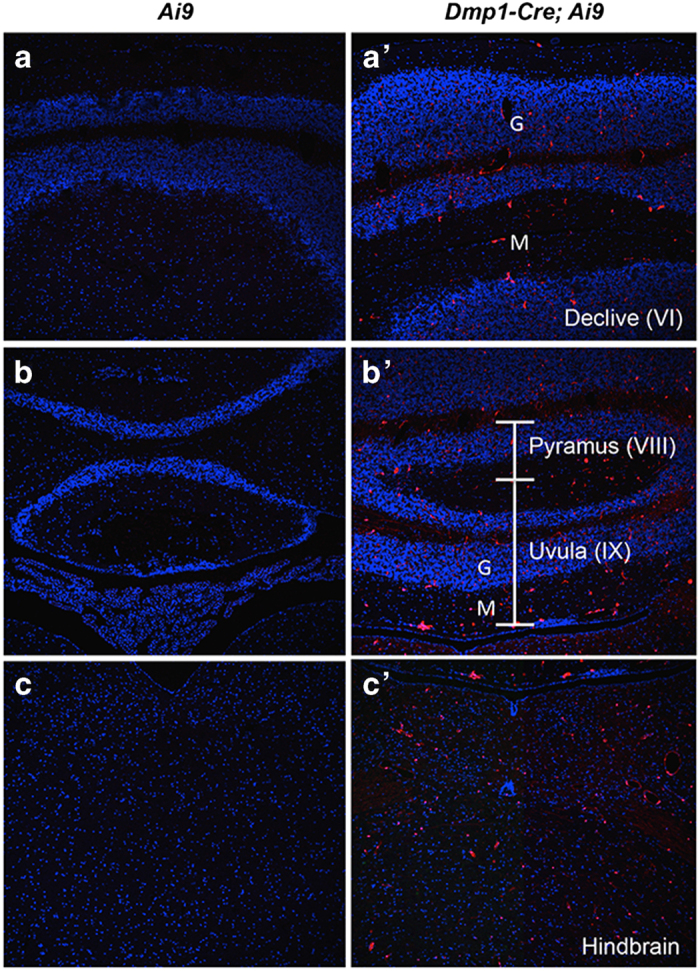
*Dmp1-Cre* targets cells in the cerebellum and the hindbrain. Direct fluorescence of tdTomato on coronal sections of the cerebellum (**a**-**b**’) and the hindbrain (**c**,**c**’). G, representative granular layer; M, representative molecular layer. Brain anatomy based on Allen Mouse Brain Atlas.

**Figure 5:**
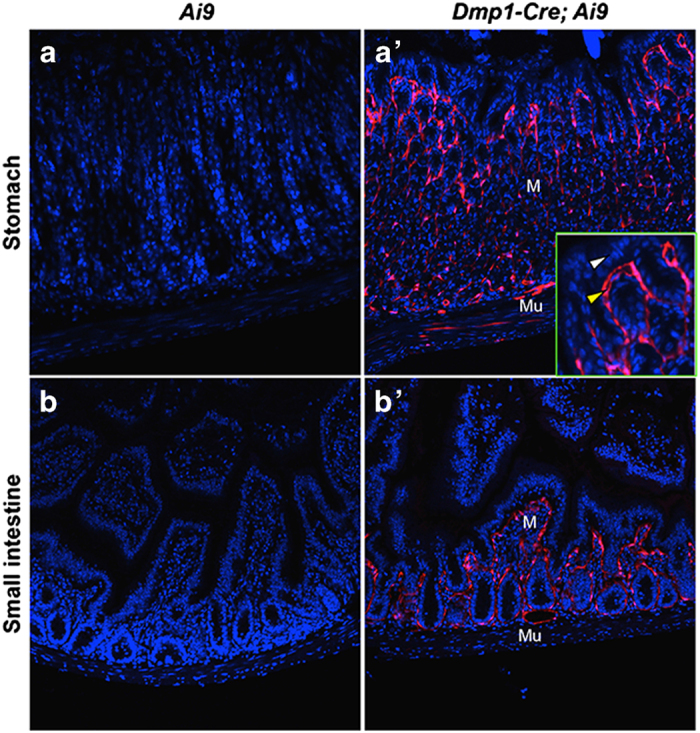
*Dmp1-Cre* targets lamina propria of the stomach and the small intestine. Direct fluorescence of tdTomato on cross-sections of the stomach (**a**,**a**’) and the small intestine (**b**,**b**’). M, mucosa; Mu, muscle wall. Inset in (**a**’) shows higher magnification of a mucosa area, denoting epithelium (white arrowhead) versus mesenchyme (yellow arrowhead).

**Figure 6:**
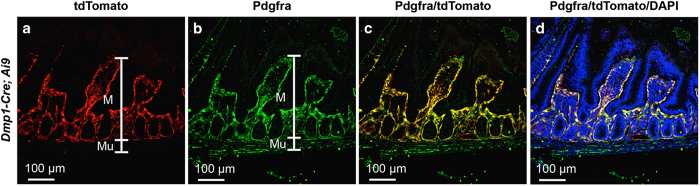
*Dmp1-Cre* targets Pdgfra-positive mesenchymal cells in the small intestine. Immunostaining against Pdgfra and direct fluorescence of tdTomato on cross-sections of the small intestine. M, mucosa; Mu, muscle wall; (**a**) tdTomato; (**b**) Pdgfra; (**c**) merge of tdTomato and Pdgfra; (**d**) merge of tdTomato, Pdgfra and DNA staining by DAPI.

We next examined other tissues of the *Dmp1-Cre*; *Ai9* mouse for potential targeting by *Dmp1-Cre*. No red fluorescence was detected in the liver, the spleen, or the gonadal fat depot. Coronal sections of the head through the parietal bone revealed a small number of red cells throughout the cerebellum and the hindbrain (Figure 4). The positive cells were present in both the molecular and granular layers of the cerebellum but did not present a specific distribution pattern; their identity was not pursued in the present study. In the stomach and the small intestine, *Dmp1-Cre* targeted many cells within the lamina propria of the mucosa (Figure 5). Co-immunostaining experiments identified the targeted intestinal cells as mesenchymal cells expressing Pdgfra (Figure 6). Interestingly, although Pdgfra-positive cells were also present in the muscle wall, *Dmp1-Cre* targeted nearly exclusively those within the laminar propria of the mucosa. Thus, *Dmp1-Cre* may be a useful tool for studying gene function in the mesenchyme of the gut mucosa.

### Deletion of *Bmpr1a* in gastrointestinal mesenchyme results in polyposis

**Figure 7:**
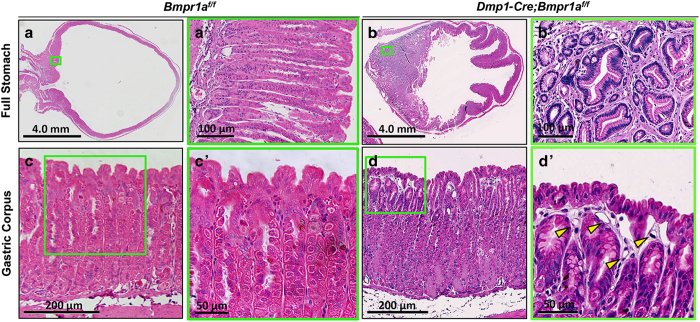
*Dmp1-Cre Bmpr1a^f/f^* mice develop gastric hyperplasia and polyps. (**a**,**b**) H&E staining of sections through the entire stomach of control (*Bmpr1r^f/f^*) (**a**) versus CKO (*Dmp1-Cre*; *Bmpr1a^f/f^*) (**b**) littermate mice at 5 weeks of age. (**a**’,**b**’) Boxed areas in **a** and **b**, respectively, shown at higher magnification. (**c**,**d**) Gastric units in the gastric corpus of control (**c**) versus CKO (**d**) littermate mice. (**c**’,**d**’) Boxed areas in (**c**,**d**) shown at higher magnification. (**d**’), long stretches of surface pit cells with no opening into gastric units. Yellow arrowheads denote increased prominence of capillaries. H&E, hematoxylin and eosin.

**Figure 8:**
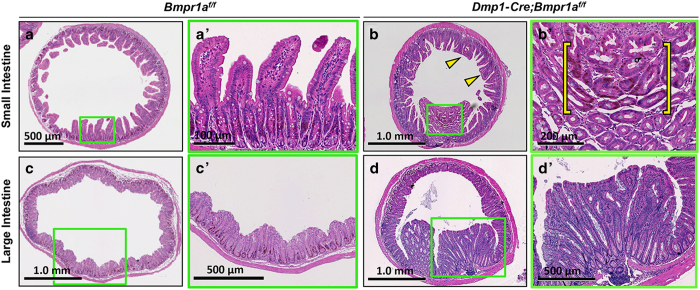
*Dmp1-Cre Bmpr1a^f/f^* mice develop intestinal polyps. (**a**–**d**) H&E staining of cross-sections through small (**a**,**b**) or large intestines (**c**,**d**) of control (*Bmpr1a^f/f^*) (**a**,**c**) or CKO (*Dmp1-Cre*; *Bmpr1a^f/f^*) (**b**,**d**) littermate mice. Yellow arrowheads in **b** denote fusing and blunting of villi. (**a**’–**d**’) Higher-magnification images of boxed areas in (**a**–**d**), respectively, showing a polyp in small (**b**’) or large (**d**’) intestine. Yellow bracket in (**b**’) denotes a pocket of proliferating epithelial cells stained brown for BrdU labeling. H&E, hematoxylin and eosin.

BMP signaling has been shown to have critical roles in development and maintenance of the gastrointestinal tract, but the significance of BMP signaling within the mesenchyme has not been demonstrated. The highly efficient targeting of the gastrointestinal mesenchyme by *Dmp1-Cre* prompted us to analyze the gut of mice with the genotype of *Dmp1-Cre*; *Bmpr1a^f/f^* (CKO). The CKO mice presented a notably higher incidence of rectal prolapse than the control littermates after 3 months of age, indicating abnormalities of the gastrointestinal tract. Histological analyses of the stomach at 5 months of age revealed that the CKO mice developed large polyps in the gastric antrum (Figure 7b and b’), whereas the *Bmpr1a^f/f^* littermate exhibited no abnormality as expected (Figure 7a and a’). These polyps contain both epithelial and mesenchymal cells, with largely unremarkable morphology but occasional foci with mild nuclear crowding and hyperplasia (Figure 7b’). In the corpus, the CKO mice exhibited a mild pit/foveolar cell hyperplasia with expansion of surface cells relative to glandular invaginations (Figure 7d’). Examination of the intestine revealed large polyps in the CKO mice, typically with 20+ polyps in the small intestine and 50+ in the large intestine, some as large as 2 mm in diameter. The polyps appeared largely hamartomatous, composed of a mixture of unremarkable epithelial, mesenchymal, and immune cells (Figure 8). Some polyps in the small intestine also contained foci harboring highly proliferative epithelium (Figure 8b’, bracket). Surveying of the CKO mice at different ages revealed that the gastrointestinal phenotype was fully penetrant by 33 days of age (*n*=4) but not at 14 days. Thus, Bmpr1a signaling in the mesenchyme is critical for maintaining the integrity of the gastrointestinal tract in postnatal mice.

## Discussion

The current study has three principal findings. First, *Dmp1-Cre* targets the osteoblast lineage starting at the preosteoblast stage, considerably earlier than previously believed. This result confirms our previous observation by using the mT/mG reporter mouse.^20^ Second, *Dmp1-Cre* also targets several non-skeletal tissues, including the skeletal muscle as previously noted, and the mesenchyme of the gastrointestinal mucosa as demonstrated here for the first time.^11^ Finally, by exploiting the unintended targeting in the gut, the study clarifies that deletion of the Bmpr1a receptor only in the mesenchyme of the gastrointestinal tract without affecting the epithelium is sufficient to cause hamartomatous polyps. Overall, *Dmp1-Cre* joins Osx-Cre as another example for a bone-targeting Cre line to possess additional activities.^22^ The studyfurther underscores the importance of assessing unintended recombination activities of many Cre strains.^23^

Although both *Osx-Cre* and *Dmp1-Cre* target preosteoblasts, osteoblasts, and osteocytes, they show important differences in other aspects. Although *Osx-Cre* marks a majority of the bone marrow stroma, *Dmp1-Cre* targets a relatively small number of cells in the bone marrow.^22,24^ The Dmp1-targeted cells are near the blood vessels and appear to be stromal cells in nature. Others have recently shown that most of the Dmp1-targeted cells in the marrow represent a subset of the Cxcl12-abundant reticular cells.^25^ The two Cre lines also show differences in the gastrointestinal tissues. Although *Osx-Cre* marks the epithelium of the mucosa in a mosaic fashion, *Dmp1-Cre* labels all of the Pdgfra-positive mesenchymal cells within the lamina propria. Distinct from *Osx-Cre*, *Dmp1-Cre* also targets all skeletal muscle fibers, as well as certain cells in the cerebellum and the hindbrain, likely reflecting the endogenous DMP1 expression in those tissues as previously reported.^9^ Similarly, *Dmp1-Cre* activity in the stomach and the intestines is consistent with endogenous Dmp1 expression, as documented by the Human Protein Atlas (http://www.proteinatlas.org/). Thus, identification of a regulatory sequence truly specific to osteocytes would require further dissection of the Dmp1 promoter. Among the other organs expressing DMP1, we did not detect Cre activity in the liver but did not examine the kidney or the pancreas.^9^ It should be noted that the efficiency of Cre recombination might vary between *Ai9* and the floxed gene of interest. The actual deletion efficiency of the targeted gene ought to be determined in a gene-specific manner. Nonetheless, we have presented a clear example that *Dmp1-Cre* can be used effectively to delete *Bmpr1a* in the gastrointestinal mesenchyme. Thus, the relevance of the other targeted cell types besides osteocytes should be considered when *Dmp1-Cre* is used in genetic studies.

BMP signaling has critical roles in normal development and maintenance of the gastrointestinal system. Loss of function mutations in *Bmpr1a* is a major cause for juvenile polyposis in patients.^26^ Here we show that deletion of *Bmpr1a* with *Dmp1-Cre* in the mouse results in the formation of numerous polyps throughout the intestine; the phenotype is reminiscent of those caused by overexpression of noggin from the villin promoter.^14^ This similarity, together with the lack of polyposis when *Bmpr1a* was deleted in the epithelium, argues that the effect seen from the villin-driven Noggin may largely stem from the inhibition of BMP signaling in the mesenchyme instead of the epithelium as originally believed.^16^ Overall, the study provides evidence that BMP signaling within the mesenchyme by itself is critical for proper gastrointestinal maintenance.
